# Two nucleotide second messengers regulate the production of the *Vibrio cholerae* colonization factor GbpA

**DOI:** 10.1186/s12866-015-0506-5

**Published:** 2015-08-19

**Authors:** Ankunda T. Kariisa, Alyssa Grube, Rita Tamayo

**Affiliations:** Department of Microbiology and Immunology, University of North Carolina Chapel Hill, 125 Mason Farm Rd, 6th Floor, Chapel Hill, NC 27599 USA; Biology Department, Juniata College, Huntingdon, PA USA

## Abstract

**Background:**

The nucleotide second messengers cAMP and c-di-GMP allow many bacteria, including the human intestinal pathogen *Vibrio cholerae*, to respond to environmental stimuli with appropriate physiological adaptations. In response to limitation of specific carbohydrates, cAMP and its receptor CRP control the transcription of genes important for nutrient acquisition and utilization; c-di-GMP controls the transition between motile and sessile lifestyles often, but not exclusively, through transcriptional mechanisms. In this study, we investigated the convergence of cAMP and c-di-GMP signaling pathways in regulating the expression of *gbpA*. GbpA is a colonization factor that participates in the attachment of *V. cholerae* to N-acetylglucosamine-containing surfaces in its native aquatic environment and the host intestinal tract.

**Results:**

We show that c-di-GMP inhibits *gbpA* activation in a fashion independent of the known transcription factors that directly sense c-di-GMP. Interestingly, inhibition of *gbpA* activation by c-di-GMP only occurs during growth on non-PTS dependent nutrient sources. Consistent with this result, we show that CRP binds to the *gbpA* promoter in a cAMP-dependent manner *in vitro* and drives transcription of *gbpA in vivo*. The interplay between cAMP and c-di-GMP does not broadly impact the CRP-cAMP regulon, but occurs more specifically at the *gbpA* promoter.

**Conclusions:**

These findings suggest that c-di-GMP directly interferes with the interaction of CRP-cAMP and the *gbpA* promoter via an unidentified regulator. The use of two distinct second messenger signaling mechanisms to regulate *gbpA* transcription may allow *V. cholerae* to finely modulate GbpA production, and therefore colonization of aquatic and host surfaces, in response to discrete environmental stimuli.

**Electronic supplementary material:**

The online version of this article (doi:10.1186/s12866-015-0506-5) contains supplementary material, which is available to authorized users.

## Background

Nucleotide second messengers in bacteria play a key role in relaying information from the extracellular environment to intracellular effectors, resulting in an adaptive physiological response. Production of these intracellular nucleotides, which include c-di-GMP, c-di-AMP, cGMP, cAMP, and (p)ppGpp, regulates fundamental processes relating including biofilm formation, motility, nutrient acquisition, stress responses, sporulation and, in some pathogens, virulence factor production (reviewed in [1–4]).

C-di-GMP, utilized by many Gram-negative and Gram-positive bacteria, usually regulates transitions between motile and non-motile lifestyles. Specifically, c-di-GMP negatively regulates flagellar motility by inhibiting flagellum biosynthesis or function, and positively regulates adherence and biofilm development in many species [5]. The intracellular levels of c-di-GMP are regulated by the opposing activities of diguanylate cyclase (DGC) biosynthetic enzymes and phosphodiesterase (PDE) hydrolytic enzymes [6–12]. Many organisms contain numerous genes that encode proteins with known or predicted functions as DGC and/or PDE enzymes. To date there are several known intracellular c-di-GMP receptors, including protein and RNA-based sensors. Proteins that directly sense c-di-GMP include transcription factors, proteins containing the well-characterized PilZ domain, and others such as catalytically inactive PDEs that retain the capacity to bind c-di-GMP [13]. In addition, two distinct classes of c-di-GMP sensing riboswitch, broadly present in bacterial genomes, have been identified [14–17]. The presence of numerous c-di-GMP metabolic enzymes and an array of intracellular c-di-GMP receptors points to complex c-di-GMP signaling networks that modulates a wide array of cellular functions.

In the human diarrheal pathogen *Vibrio cholerae*, c-di-GMP promotes biofilm formation and inhibits flagellar motility [18–20]; in addition, c-di-GMP inhibits the expression of virulence genes including those encoding the major virulence factor, cholera toxin [21]. To date, three transcription factors that directly sense c-di-GMP have been identified in *V. cholerae*: VpsT, VpsR and FlrA [22–24]. Each of these was previously characterized as a regulator of biofilm formation and/or motility [20, 25–28]. Five proteins containing a PilZ domain have been described in *V. cholerae*, some of which have roles in biofilm, motility and/or virulence [29]. The *V. cholerae* genome also encodes two c-di-GMP riboswitches, Vc1 and Vc2, which lie upstream of *gbpA* and VC1722*,* respectively [14].

cAMP signaling is widespread in bacteria, and intracellular cAMP levels are heavily influenced by the availability of extracellular nutrient sources [30]. In Gram-negative bacteria, uptake of a preferred sugar by the phosphoenolpyruvate-carbohydrate phosphotransferase transport system (PTS) relies on a sugar-specific transporter, EIIA. Following uptake of a PTS sugar, EIIA and other PTS components participate in a phosphorylation cascade that culminates phospho-transfer from EIIA to the incoming sugar. The phosphorylation state of EIIA serves as a measure of PTS sugar availability. Under conditions of PTS sugar limitation (and absence of a PTS sugar substrate), EIIA remains phosphorylated and stimulates the adenylate cyclase (AC), triggering cAMP biosynthesis. To date, the only known sensor of cAMP in bacteria is the cAMP receptor protein (CRP) [30]. Together, the cAMP-CRP complex promotes the expression of genes involved in the uptake and utilization of non-PTS nutrient sources.

*V. cholerae* uses the cAMP-CRP system to respond to the absence of PTS-dependent sugars such as *N*-acetylglucosamine, sucrose, mannitol and fructose [31]. Unlike *E. coli*, in which glucose utilization is PTS-dependent, glucose can be utilized via PTS and non-PTS mechanisms in *V. cholerae* [31]. *V. cholerae* has also adapted the cAMP-CRP pathway to promote motility and inhibit biofilm formation [32, 33]. Transcriptome analyses have shown that *cya* and *crp* deletion mutants (which lack cAMP biosynthesis and sensing, respectively) have increased expression of genes involved in extracellular matrix production, reduced expression of genes involved in flagellum biosynthesis and chemotaxis [32, 33]. In addition, a *crp* deletion mutant has reduced expression of genes important for colonization and is attenuated in a mouse model of infection [32]. Interestingly, the cAMP-CRP signaling pathway impinges upon the c-di-GMP signaling network; a *crp* mutant has altered expression of numerous genes involved in c-di-GMP metabolism [33].

*V. cholerae* naturally inhabits aquatic reservoirs, where it associates with chitinous surfaces such the exoskeletons of zooplankton and crustaceans [34]. *V. cholerae* can be ingested through consumption of contaminated food or water and subsequently colonize the small intestine and cause diarrheal disease in humans. The colonization factor GbpA aids in the attachment of *V. cholerae* to surfaces in its native aquatic environment and in the host intestine [35, 36]. GbpA recognizes N-acetylglucosamine (GlcNAc), a component of mucin and a modification of glycoprotein and lipids found on the surface of the intestinal epithelium, thus mediating interactions with the host intestinal epithelium [35, 36]. GlcNAc also comprises the polymer chitin, a major component of the exoskeletons of crustaceans and zooplankton, thus serving as a substratum for *V. cholerae* colonization in aquatic reservoirs [35]. In addition to serving as a ligand for GbpA, GlcNAc also regulates *gbpA* expression via the transcriptional regulator NagC [37, 38]. In *V. cholerae* and some other bacterial species, the transcription factor NagC regulates gene expression in response to GlcNAc and typically controls genes involved in GlcNAc uptake and metabolism [38–42]. Transcriptional profiling of a *V. cholerae nagC* mutant indicated that NagC represses *gbpA* transcription [38], suggesting that *gbpA* expression is down-regulated in the presence of mucin- and chitin-derived GlcNAc in the small intestine and in the aquatic environment, respectively [36–38].

In this study, we investigated the combined roles of the second messengers cAMP and c-di-GMP in regulating *gbpA* transcription. We report that c-di-GMP inhibits *gbpA* transcription independently of the previously described c-di-GMP riboswitch. Expression of *gbpA* is also influenced by the availability of PTS-dependent carbohydrates, and is accordingly regulated by cAMP-CRP through direct binding of this complex to the *gbpA* promoter. Together, our results indicate that *gbpA* transcription is regulated by both c-di-GMP and cAMP, in opposing fashion. The c-di-GMP and cAMP second messenger signaling pathways may thus function together to modulate the production of GbpA in response to discrete extracellular stimuli, likely impacting the ability of *V. cholerae* to colonize GlcNAc-containing surfaces in the aquatic and host environments.

## Methods

### Growth conditions and media

*Escherichia coli*, *V. cholerae* C6706 and mutant derivatives were cultured at 37 °C with aeration in Luria-Bertani (LB) broth containing 100 μg/ml streptomycin (Sm), 10 μg/ml chloramphenicol (Cm), and/or 50 μg/ml ampicillin (Amp), as appropriate. Where specified, *V. cholerae* was grown in M9 minimal medium (Fisher Scientific) supplemented with trace metals (1 ml l^−1^ of 5 % MgSO_4_, 0.5 % MnCl_2_4H_2_O, 0.5 % FeCl_3_, 0.4 % nitrilotriacetic acid) [43] and 0.5 % (w/v) of the indicated carbon source.

### Strain construction

Strains and plasmids included in this study are listed in Additional file 1: Table S1. Primer information is contained in Additional file 1: Table S2. The *gbpA*, *nagC*, *crp*, *cpdA, cya, vpsT, vpsR and flrA* genes were mutated by standard allelic exchange methods. A deletion in the Vc1 sequence upstream of *gbpA* was deleted in a similar manner. Using genomic DNA from *V. cholerae* C6706 as template, ~800 bp fragments upstream and downstream of the sequences to be deleted were amplified by PCR using primers named according to the pattern geneF1 + geneR1 for the upstream region of homology and geneF2 + geneR2 for the downstream region of homology. The primers introduced restriction sites (underlined sequences in Table S2) allowing ligation of the resulting fragments to each other and into the pCVD442 suicide vector. The exception is the *vpsR* mutation, for which the two fragments were joined by splicing by overlap extension, and then ligated into pCVD442. The ligations were transformed into DH5αλpir by electroporation and transformed colonies were identified on LB-Amp agar. The desired clones containing the upstream and downstream fragments were identified by PCR using primers geneF1 + geneR2 and/or pCVDseqF + pCVDseqR, which flank the multiple cloning site of pCVD442. The allelic exchange steps were done as described previously [44]. Colonies were screened for the desired deletion by PCR using the corresponding geneF0 + geneR2 primers.

VC1592, which encodes an EAL domain c-di-GMP phosphodiesterase [45, 46], was amplified from C6706 genomic DNA using primers VC1592eF + VC1592eH6R. The product was digested with SacI and PstI, ligated into similarly digested pBAD33, and transformed into DH5α. Cm-resistant transformants were screened for the desired insert by PCR, and isolates were confirmed by sequencing.

The pP*gbpA*-Vc1-*lacZ* and pP*gbpA*-∆Vc1-*lacZ* plasmids were constructed by amplifying the *gbpA* promoter and 5′ UTR from *V. cholerae* C6706 and ΔVc1 genomic DNA, respectively, by PCR using gbpAP2F + gbpAR2. The PCR products were digested with *Eco*RI and *Sal*I and ligated into pP*lac*thiM#2-*lacZ* [47] digested with the same enzymes. The ligation reaction was transformed into DH5α cells by electroporation, and Amp-resistant colonies obtained were screened with primers pLacSeq + gbpAR2. The resulting plasmids have P*gbpA*-UTR (wild type or ∆Vc1) as a translational fusion to *lacZ*, with the *lac* promoter driving transcription. The plasmids were introduced by electroporation into *V. cholerae* C6706 in which the endogenous *lacZ* gene was inactivated [48].

The pBAD33, pPDE (pBAD33::*vieA*), pPDE^mut^ (pBAD33::*vieA-E170A*), and pVC1592 (pBAD33::VC1592) plasmids were introduced into the indicated strains by electroporation. Cm-resistant isolates were confirmed to have the desired plasmid by PCR.

### Manipulation of the intracellular c-di-GMP level in *V. cholerae*

The intracellular c-di-GMP level was depleted through the ectopic expression of a *V. cholerae* c-di-GMP phosphodiesterase gene, *vieA*, as described previously [49, 50]. Briefly, *V. cholerae* bearing pBAD33 (“vector”) or pBAD33::*vieA* (“pPDE”) were grown in 2 ml LB-Sm-Cm broth at 37 °C with aeration. Ampicillin was included at 50 μg/ml if the strains also contained a *lacZ* reporter plasmid. For the indicated experiments, *V. cholerae* with pPDE^mut^ (encoding enzymatically inactive VieA PDE) or pVC1592 (encoding an alternative c-di-GMP PDE) were included [45]. At early exponential phase (OD_600_ ~ 0.2), 0.2 % L-arabinose was added to the cultures to induce *vieA* transcription from the P_*BAD*_ promoter, and incubations were continued at 37 °C with aeration. Samples were collected at mid-exponential phase (optical density at 600 nm (OD_600_) ~ 0.5-0.7) for western blotting or qRT-PCR analysis as described below.

### Reporter activity assays

The β-galactosidase activity was measured for *V. cholerae* strains containing the P_*gbpA*_-Vc1-*lacZ* fusion, with vector (pBAD33) or pPDE. Strains were grown in 2 ml M9 minimal medium with 0.5 % (w/v) glucose, maltose, sucrose, fructose or casamino acids. Ampicillin (50 μg/ml) and/or chloramphenicol (10 μg/ml) were included in the media as appropriate to maintain plasmids. Cultures were incubated at 37 °C with aeration. Expression of the *vieA* PDE gene was induced using 0.2 % L-arabinose as described above. Samples were grown to mid-exponential phase (OD_600_ 0.45-0.6), and 100 μl were assayed for hydrolysis of ortho-nitrophenyl-β-D-galactoside using a Miller assay [51]. At least three independent experiments were done, and the data were combined. Statistical analyses were done using unpaired t-tests.

### GbpA antibody production

Anti-GbpA antiserum was produced by Yenzym 192 Antibodies, LLC, South San Francisco, CA. Antiserum was raised in rabbits to a synthetic peptide (CSNATQYQPGTGSHWEMAWDKR) that corresponds to GbpA from *V. cholerae*. The animal facilities were NIH/OLAW/PHS assured, USDA certified, and IACUC regulated.

### GbpA and CRP detection by western blot

Equal-volume samples of mid-exponential phase cultures, normalized to OD_600_ (0.45-0.6), were collected. For detection of GbpA, the samples were centrifuged to remove the bacteria. Supernatant proteins were TCA precipitated, separated by electrophoresis, and subjected to western blotting with rabbit anti-GbpA antibodies. For detection of CRP, cells grown as above were collected by centrifugation. Whole lysates were electrophoresed and transferred to nitrocellulose membranes. CRP (~23.6 kDa) was detected using anti-CRP monoclonal antibodies (Neoclone). For cell lysates, RNA Polymerase β subunit (~150 kDa) served as a loading control and was detected with monoclonal antibodies (AbCam). In all western blots, goat α-rabbit IgG conjugated with IR800 dye (Thermo Scientific) was used as the secondary antibody. The blots were imaged using an Odyssey imaging system (LI-COR). At least three independent experiments were done, and a representative image is shown. Densitometry analyses were done using the Odyssey software. The intensities of the bands corresponding to GbpA in supernatants were normalized to those of a cross reactive band (indicated by asterisks in relevant images). For CRP quantification, the intensities of the bands of CRP in lysates were normalized to those of RNAP.

### RNA purification and analysis using quantitative real-time PCR

Transcriptional analyses using quantitative reverse-transcriptase PCR (qRT-PCR) were done as previously described [52]. Briefly, RNA was purified from mid-exponential phase (OD_600_ ~ 0.5-0.7) cultures. Genomic DNA was removed using the TURBO DNA-free kit (Ambion). For cDNA synthesis, RNA (200 ng) was reverse transcribed using the Tetro cDNA Synthesis Kit (Bioline). We included control reactions without reverse transcriptase for every cDNA sample. For the real time PCR reaction, cDNA and control samples were combined with 2× SYBR/fluorescein mix (SensiMix; Bioline) and 7.5 μM of each primer (named according to the scheme gene-qF and gene-qR for forward and reverse primers, respectively, Additional file 1: Table S2). We used the following program to amplify target cDNA: 95 °C for 10 min, followed by 40 cycles of 95 °C for 30 s, 55 °C for 1 min, and 72 °C for 30 s. Melt curves were included to verify amplification of single products. The data were analyzed using the ΔΔCt method, with Ct values normalized to the specified reference strain/condition, and to the Ct values of the reference gene *rpoB* and/or *gyrA* in each sample [52–54]. For each strain/condition, a minimum of three independent samples was tested. Statistical significance was determined by unpaired *t*-test.

### Cloning and expression of *crp* and purification of the recombinant protein

The CRP gene was amplified from *V. cholerae* C6706 genomic DNA using primers CRPeF + CRPeH6R, which incorporate EcoRI and SalI restriction sites into the product, respectively. The CRPeH6R primer introduces 6 histidine codons at the 3′ end of the gene. The PCR product was digested with EcoRI and SalI and ligated into similarly digested pMMB67EH, a low-copy vector allowing IPTG-inducible gene expression [55]. Ligations were transformed into *E. coli* DH5α cells. Ampicillin-resistant colonies containing pMMB67EH with *crp-his6* insert were identified by PCR, yielding pMMB::*crp*-his6.

*E. coli* DH5α containing pMMB::*crp*-his6 was grown in LB broth at 37 °C with aeration to early exponential phase (OD_600_ ~ 0.2), at which point IPTG was added for a 0.5 mM final concentration. The culture was grown at 37 °C with aeration to mid- exponential phase (OD 600 nm ~ 0.7), then cells were collected by centrifugation. Cells were suspended in His6 lysis buffer consisting of 10 mm Tris, pH 8, 300 mm NaCl, 50 mm NaH_2_PO_4_, 10 % glycerol, 1 mm phenylmethylsulfonyl fluoride, and 5 mM imidazole [10]. The cells were lysed by sonication, and CRP was purified by affinity chromatography with Ni-NTA resin (ThermoFisher) using the general methods described previously [10, 50]. The eluates were analyzed by SDS-PAGE and coomassie staining. CRP-containing fractions were dialyzed against 5 mM Tris 8.0, 10 mM MgCl2, 5 mM KCl, 5 mM CaCl2, 10 % glycerol using 10,000 MWCO Slide-A-Lyzer Dialysis Cassettes (Thermo Scientific). The CRP preparations were estimated to be > 95 % pure. Glycerol was added to a final concentration of 20 %, and the protein concentration was determined using the BCA Protein Assay Kit (Pierce). Aliquots of the protein were stored at −20 °C.

### Electrophoretic mobility shift assays (EMSAs)

A 293 base pair *gbpA* promoter fragment was amplified from *V. cholerae* C6706 genomic DNA using primers gbpAP2F + Vc1R2. A 133 base pair non-specific control DNA fragment internal to the *gbpA* ORF was amplified with gbpAqF2 + gbpAqR2. EMSAs were done as previously described [56]. Binding reactions were done in 10 mM Tris pH 7.5, 1 mM EDTA, 100 mM KCl, 0.1 mM DTT, 5 % glycerol (v/v) and 0.01 mg/ml BSA (final concentrations). The *gbpA* promoter fragment, CRP, and cAMP (Sigma-Aldrich) were used at final concentrations of 2.5 ng/μl, 11 ng/μl, and 33 μM, respectively. C-di-GMP (Biolog) was used at 33 μM, or 333 μM when in competition with cAMP. The non-specific DNA fragment (75 ng) was added to every mixture. After 1 h incubation at room temperature (~25 °C), the samples were electrophoresed on a 6 % TAE polyacrylamide gel. The gel was stained with GelRed (Biotium, Inc.) in TAE for 10 min, and then visualized under UV light.

## Results

### Low c-di-GMP induces *gbpA* transcription

A putative c-di-GMP riboswitch, named Vc1, was previously predicted upstream of the *gbpA* open reading frame [14]. The Vc1 element shares 75 % identity with the well-characterized c-di-GMP riboswitch Vc2, which has been shown to bind c-di-GMP directly and positively regulate expression of a reporter gene in response to c-di-GMP in a heterologous bacterial host [14]. The identification of the putative c-di-GMP riboswitch Vc1 upstream of *gbpA* suggests that *gbpA* expression is regulated by this signaling molecule. To test this, we used a previously described strategy for manipulating intracellular c-di-GMP levels in *V. cholerae*: the ectopic expression of a c-di-GMP metabolism gene. The expression of the *vieA* gene, which encodes a c-di-GMP phosphodiesterase, has been used to artificially manipulate intracellular c-di-GMP in *V. cholerae*, resulting in phenotypes consistent with reduced c-di-GMP: increased motility, reduced biofilm formation, and increased cholera toxin production [21, 50, 57]. To establish that *vieA* expression similarly results in altered intracellular c-di-GMP in *V. cholerae* C6706, the wild type strain used in this study, we evaluated the effect of *vieA* expression from plasmid “pPDE” on motility and biofilm formation, which are inhibited and promoted by c-di-GMP in *V. cholerae*, respectively. To assay swimming motility, colonies of *V. cholerae* C6706 with vector (pBAD33) or pPDE were inoculated into soft agar medium containing 0.2 % L-arabinose to induce gene expression, and expansion from the inoculation point was monitored. *V. cholerae* C6706 with pPDE showed significantly increased motility compared to the vector control (Additional file 2: Figure S1A). Importantly, *V. cholerae* C6706 with pPDE^mut^, which contains an E170A mutation that renders VieA enzymatically inactive [10], showed motility equal to the vector control. These results indicate that c-di-GMP hydrolysis by VieA augments *V. cholerae* motility, consistent with previous observations [57]. In addition, expression of another EAL-domain c-di-GMP PDE gene, VC1592, enhanced motility comparably to *vieA*, indicating that the effect on motility is not specific to the VieA PDE [45, 46]. To assay biofilm formation, *V. cholerae* C6706 with vector or pPDE were grown statically for 24 h in LB broth containing 0.2 % L-arabinose for gene induction, and then the biofilm biomass was evaluated by crystal violet staining. Biofilm formation of *V. cholerae* C6706 with pPDE was reduced to 25 % of that generated by the vector control strain; expression of VC1592 similarly reduced biofilm production (Additional file 2: Figure S1B). Biofilm formation of *V. cholerae* C6706 with pPDE^mut^ was intermediate between *V. cholerae* C6706 strains expressing functional PDE genes and the vector control strain. Combined with the motility assay results, these findings indicate that ectopic production of VieA effectively reduces intracellular c-di-GMP in *V. cholerae* C6706, reflected by increased swimming motility and reduced biofilm formation.

**Fig. 1:**
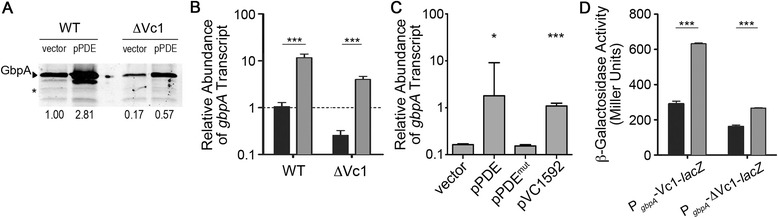
c-di-GMP inhibits *gbpA* expression independently of the Vc1 riboswitch. **a** GbpA production by *V. cholerae* wild type and ∆Vc1 strains, each with vector (pBAD33) or pPDE, was determined by western blot. PDE gene expression was induced as described in the Methods. The image shown is a representative of three independent experiments. Densitometry analyses were done by normalizing the intensities of the bands corresponding to GbpA to the intensities of a cross-reactive band in the same lane (indicated by an asterisk), then comparing the normalized value to that of wild type *V. cholerae* with vector only. The fold change relative to wild type is indicated below each lane. **b** qRT-PCR was used to measure *gbpA* transcript levels in the strains described in (A), in *V. cholerae* with vector (black bars) or with pPDE (grey bars). The data were normalized relative to the wild-type containing vector only, using *rpoB* as the reference gene. **c** P_*gbpA*_-Vc1-*lacZ* or P_*gbpA*_-∆Vc1-*lacZ* fusions were each introduced into *V. cholerae* with vector (black bars) or pPDE (grey bars), and the β-galactosidase activity in culture lysates of these strains was measured. **d** qRT-PCR was used to measure *gbpA* transcript levels in *V. cholerae* with vector, pPDE, pPDE^mut^ or pVC1592. The data were normalized relative to the wild-type containing vector, using *rpoB* as the reference gene. **b**-**d** Shown are the mean values and standard deviations of at least three independent experiments. ***P < 0.001 by unpaired *t*-test comparing the indicated sets of data

Using this expression strategy to manipulate intracellular c-di-GMP, we evaluated the effect of altering c-di-GMP on the abundance of the *gbpA* transcript and GbpA protein in *V. cholerae* with pPDE compared to *V. cholerae* with vector (grown in the presence of 0.2 % L-arabinose to drive *vieA* transcription as described in the Methods). Western blot analysis of GbpA production showed that GbpA was 2.8-fold more abundant in *V. cholerae* with reduced c-di-GMP (pPDE) (Fig. 1a). Regulation by c-di-GMP occurred at the level of transcription, as quantitative real time PCR analysis indicated that *gbpA* transcript levels were 11.1-fold higher in *V. cholerae* with pPDE (Fig. 1b). Importantly, *gbpA* transcript was similarly increased in *V. cholerae* with pVC1592, in which the alternative c-di-GMP PDE is produced, and is not altered in *V. cholerae* with pPDE^mut^, in which the enzymatically inactive VieA is produced (Fig. 1c). These results indicate the effect of VieA production on *gbpA* levels was due to depletion of c-di-GMP, and not a consequence of another regulatory function of VieA. Throughout the remainder of the study, we used *V. cholerae* C6706 with pPDE grown in the presence of arabinose to represent low c-di-GMP conditions, with *V. cholerae* C6706 with vector grown in the same conditions serving as the wild-type c-di-GMP control.

### c-di-GMP inhibition of *gbpA* transcription is independent of the putative riboswitch Vc1

We next tested whether c-di-GMP induction of GbpA production is dependent on the putative riboswitch Vc1. To do this, we assessed the effect of depleting c-di-GMP in *V. cholerae* lacking the Vc1 sequence. This strain, ∆Vc1, lacks nucleotides 17–202 of the transcript, which encompass most of the 5′UTR including the c-di-GMP riboswitch, but retains the native *gbpA* promoter and the native ribosomal binding site. The pPDE and control plasmids were introduced into the ΔVc1 mutant, and the strains were assessed for changes in *gbpA* expression. In the ∆Vc1 strain with vector, *gbpA* transcript (Fig. 1b) and GbpA protein (Fig. 1a) were diminished by 75 and 83 %, respectively, compared to the wild type with vector, suggesting that removal of nucleotides 17–202 reduces expression. Yet depletion of c-di-GMP in the ΔVc1 strain (pPDE) increased GbpA production 3.4-fold (Fig. 1b) and *gbpA* transcription 15.4-fold (Fig. 1a), an effect equivalent to that seen in the wild type background.

In addition, we used *lacZ* fusions to either the wild type *gbpA* promoter and 5′UTR (P_*gbpA*_-Vc1-*lacZ*) or the promoter and 5′UTR lacking Vc1 (P_*gbpA*_-∆Vc1-*lacZ*) as reporters of *gbpA* expression. We measured β-galactosidase activity in *V. cholerae* with these plasmid-borne reporters, each with either vector or pPDE. In both the P_*gbpA*_-Vc1-*lacZ* and P_*gbpA*_-∆Vc1-*lacZ* reporter strains, reduction of c-di-GMP (pPDE) resulted in a statistically significant ~2-fold increase in β-galactosidase activity (Fig. 1d). Thus, c-di-GMP inhibition of *gbpA* expression is independent of Vc1, indicating that low intracellular c-di-GMP concentrations promote *gbpA* transcription initiation via the *gbpA* promoter.

### The c-di-GMP responsive regulators VpsT, VpsR and FlrA are not involved in the regulation of *gbpA* transcription by c-di-GMP

We next aimed to determine the mechanism by which c-di-GMP inhibits *gbpA* transcription. We hypothesized that a c-di-GMP sensing transcription factor interacts with the *gbpA* promoter, either inhibiting expression in response to c-di-GMP, or alleviating activation in response to c-di-GMP. We first focused on three previously identified c-di-GMP binding transcription factors, VpsT, FlrA and VpsR, as potential mediators of c-di-GMP regulation of *gbpA* expression [22–24]. Transcriptional profiling studies analyzing *V. cholerae vpsT*, *vpsR* and *flrA* mutants have implicated each of the regulators in controlling *gbpA* expression [20, 58]. The FlrA protein was recently shown to directly sense c-di-GMP, and c-di-GMP binding inhibits the interaction of FlrA with a target flagellar gene promoter (*flrBC*) [24]. Similarly, VpsT, a well-known activator of biofilm exopolysaccharide genes in *V. cholerae* [28], was recently shown to bind c-di-GMP, resulting in enhanced binding to the EPS gene promoters [22]. Finally, VpsR, another positive regulator of *V. cholerae* exopolysaccharide gene expression and biofilm production [26], appears to interact directly with c-di-GMP; unlike VpsT and FlrA, c-di-GMP binding did not influence VpsR binding to target promoters (*vpsT* and *aphA*) [23].

**Fig. 2:**
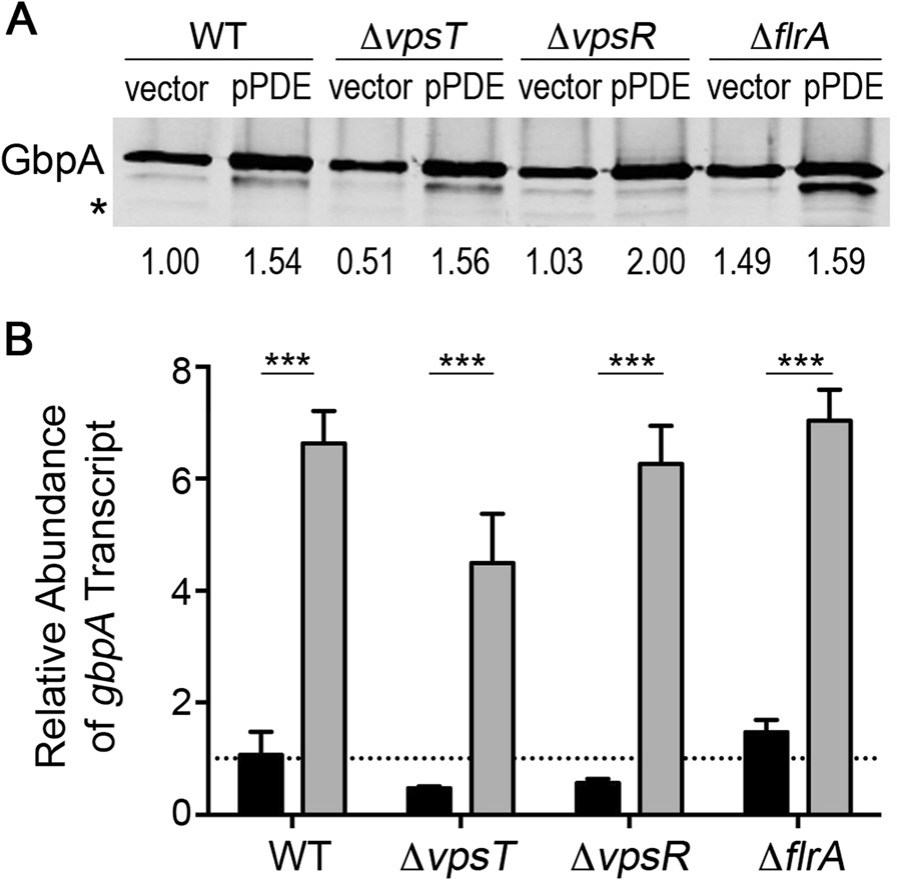
Known c-di-GMP effectors FlrA, VpsT and VpsR do not regulate *gbpA* in response to c-di-GMP. **a** GbpA levels in the supernatants of wild type *V. cholerae*, ∆*flrA,* ∆*vpsT* and Δ*vpsR* strains, each with wild type (vector) or reduced levels of c-di-GMP (pPDE), were measured by western blot. PDE gene expression was induced as described in the Methods. The image shown is a representative of three separate experiments. Densitometry analyses were done by comparing the intensities of the GbpA bands to the intensities of a cross-reactive band in the same lane (indicated by an asterisk), then normalizing the value to that of wild type *V. cholerae* with vector. The fold change relative to the wild type is indicated below each lane. **b** qRT-PCR was used to measure the *gbpA* transcript abundance in wild type, ∆*flrA,* ∆*vpsT* and Δ*vpsR* strains of *V. cholerae,* each with wild type (vector, black bars) and reduced levels of c-di-GMP (pPDE, grey bars). The data were normalized relative to the wild-type containing vector only, using *rpoB* as the reference gene. Shown are the means and standard deviations from at least three independent samples. For the indicated comparisons, ***P < 0.001 by unpaired *t*-test

We tested the effect of *vpsT, vpsR* or *flrA* mutation on *gbpA* expression in response to c-di-GMP. The pPDE and control plasmids were introduced into the Δ*vpsT,* Δ*vpsT* and Δ*flrA* mutants, and *gbpA* expression was assessed by qRT-PCR and by western blot. Transcript analysis showed that the *flrA* mutant had somewhat (1.4-fold) higher *gbpA* transcript levels, and the *vpsT* and *vpsR* mutants had lower levels (55 and 46 % decreased, respectively), than the parental strain (Fig. 2b). These differences were not statistically significant, but the trends were supported by the results of the western blots comparing GbpA protein abundance in the mutants compared to the wild type with vector (Fig. 2a). Furthermore, depletion of c-di-GMP (pPDE) in the Δ*flrA*, Δ*vpsT* and Δ*vpsR* mutants resulted in 4.8-, 9.6- and 11.0-fold increased *gbpA* transcript, respectively, compared to the mutants with unmodified c-di-GMP (Fig. 2b). Inhibition of *gbpA* expression by c-di-GMP in the mutants and the wild type was apparent at the protein level as well (Fig. 2a). The observed changes were equivalent to the increase seen in the wild type background. Thus, none of the known c-di-GMP sensing transcription factors are required for positive regulation of *gbpA* expression by depletion this second messenger. It remains possible that c-di-GMP influences *gbpA* expression via these transcription factors in more subtle ways that are not apparent using the c-di-GMP depletion strategy.

### c-di-GMP inhibits *gbpA* transcription in a nutrient dependent manner

GlcNAc and GlcNAc-containing mucin have been shown to regulate *gbpA* expression, and to date the only known transcriptional regulator of *gbpA* is NagC, a repressor of genes involved in GlcNAc utilization and of *gbpA* transcription [36–38]. We sought to determine how c-di-GMP impacts regulation of the *gbpA* promoter in the presence of N-acetylglucosamine (GlcNAc). We reasoned that GlcNAc, and perhaps other carbohydrates, may affect c-di-GMP inhibition of *gbpA* expression, and that NagC may participate in this process. Specifically, we postulated that c-di-GMP promotes NagC inhibition of *gbpA* expression, such that depletion of c-di-GMP results in NagC de-repression.

**Fig. 3:**
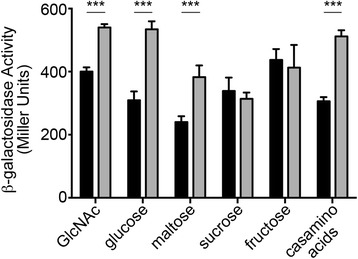
c-di-GMP inhibition of *gbpA* expression is influenced by carbon source availability. *V. cholerae* strains containing the P_*gbpA*_-Vc1-*lacZ* reporter fusion, with wild type c-di-GMP (vector, black bars) or reduced c-di-GMP (pPDE, grey bars), were grown in M9 minimal medium with 0.5 % (w/v) N-acetylglucosamine (GlcNAc), glucose, maltose, sucrose, fructose or casamino acids at 37 °C with aeration to mid-logarithmic phase. PDE gene expression was induced as described in the Methods. Transcription was measured using β-galactosidase assays. Shown are the means and standard deviations from at least three independent experiments. For the indicated comparisons, ***P < 0.001 by unpaired *t*-test

To test the effect of GlcNAc on regulation of *gbpA* expression by c-di-GMP, we generated *V. cholerae* strains with a translational fusion of *lacZ* to the *gbpA* promoter and 5′UTR, with either pPDE or the vector control. These strains were grown in a defined medium (M9 minimal medium, MM) supplemented with 0.5 % (w/v) GlcNAc, glucose, maltose, sucrose, fructose or casamino acids. The additional carbohydrates were included to address whether the potential effect of GlcNAc is specific to this sugar, and casamino acids were included as a non-carbohydrate carbon source control. The cultures were grown to mid-exponential phase (OD_600_ 0.45-0.6), and expression of *gbpA* was measured by β-galactosidase assay, allowing normalization to the optical density of the culture to adjust for differences in growth due to the carbon source. Expression of *gbpA* was variable in the media tested (Fig. 3, black bars), possibly due to differences in growth rate with the different carbon sources (Additional file 2: Figure S2). Compared to *V. cholerae* with wild type c-di-GMP levels, reduction of c-di-GMP led to significantly increased reporter activity in *V. cholerae* grown in GlcNAc (1.35-fold), glucose (1.73-fold), maltose (1.59-fold) and casamino acids (1.67-fold), but not in bacteria grown in sucrose or fructose (Fig. 3, grey bars). The results could not be attributed to growth rate, as depleting c-di-GMP (pPDE) did not affect overall growth in these media (Additional file 2: Figure S2). These results indicate that growth on GlcNAc does not interfere with c-di-GMP regulation of *gbpA* expression.

**Fig. 4:**
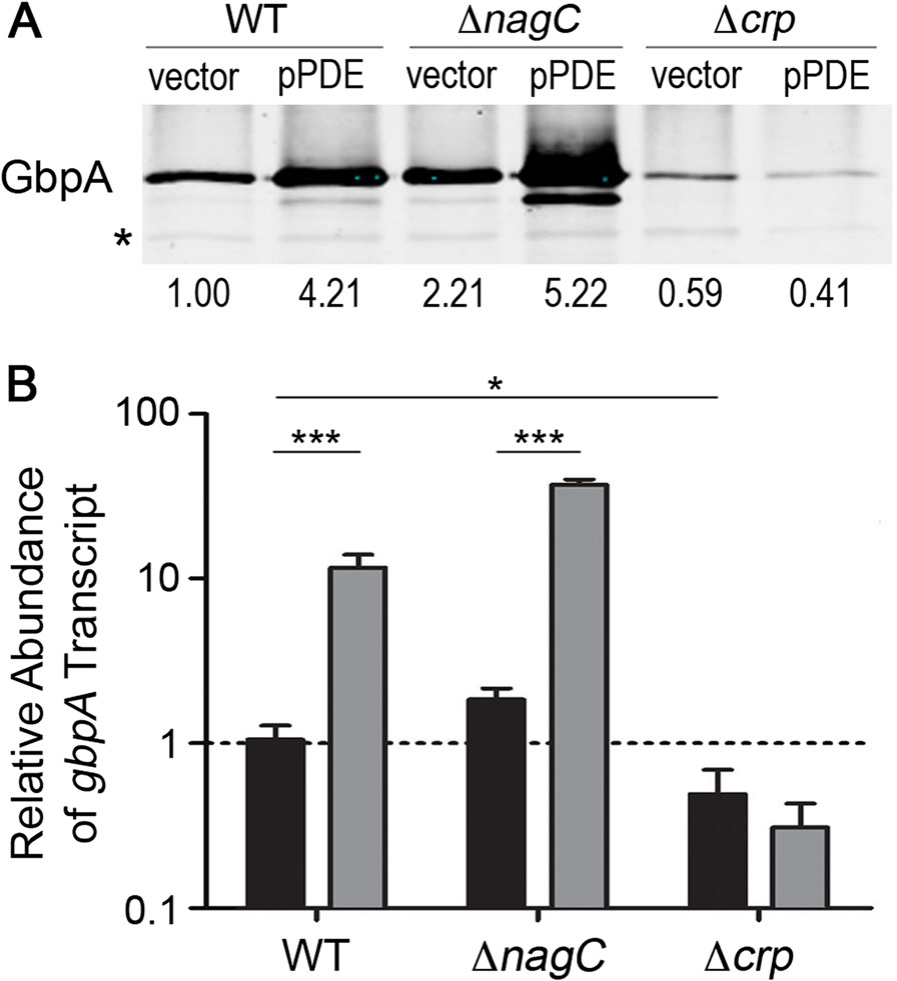
Reduction of c-di-GMP induces *gbpA* expression in a CRP dependent manner. **a** GbpA levels in the supernatants of wild type *V. cholerae*, ∆*nagC* and ∆*crp* strains, each with wild type (vector) and reduced levels of c-di-GMP (pPDE), were measured by western blot. PDE gene expression was induced as described in the Methods. The image shown is a representative of three separate experiments. Densitometry analyses were done by comparing the intensities of the GbpA bands to the intensities of a cross-reactive band in the same lane (indicated by an asterisk), then normalizing the value to that of wild type *V. cholerae* with vector. The fold change relative to the wild type is indicated below each lane. **b** qRT-PCR was used to measure the *gbpA* transcript abundance in wild type, ∆*nagC* and ∆*crp* strains of *V. cholerae,* each with wild type (vector, black bars) and reduced levels of c-di-GMP (pPDE, grey bars). The data were normalized relative to the wild-type containing vector only, using *rpoB* as the reference gene. Shown are the means and standard deviations from at least three independent samples. For the indicated comparisons, *P < 0.05, ***P < 0.001 by unpaired *t*-test

To test whether NagC is required for c-di-GMP inhibition of *gbpA* expression, pPDE and the control vector were introduced into *V. cholerae* with an in-frame deletion of *nagC* (∆*nagC*). Consistent with previous reports [37, 38], deletion of *nagC* resulted in 2.2-fold higher GbpA protein (Fig. 4a) and 1.8-fold higher *gbpA* transcript abundance (Fig. 4b) than in the wild type*.* Upon lowering c-di-GMP levels through PDE gene expression, we observed 20-fold and 2.4-fold increases in *gbpA* transcript and GbpA protein, respectively, comparable to those seen in the wild type background (Fig. 4a and 4b, respectively). Thus, NagC is not required for *gbpA* inhibition by c-di-GMP, corroborating the lack of an effect of growth with GlcNAc as the sole carbon source.

### Low c-di-GMP induces *gbpA* transcription in a cAMP-CRP-dependent manner

In *V. cholerae*, GlcNAc, sucrose and fructose are solely imported via a phosphoenolpyruvate-carbohydrate phosphotransferase system (PTS), and maltose is taken up via a PTS-independent mechanism [31]. Glucose is a PTS-substrate that can also be imported via a PTS-independent pathway, and GlcNAc has been reported to be a PTS dependent carbohydrate in *V. cholerae* [31]. However, a *V. cholerae nagE* mutant, which does not produce the GlcNAc PTS transporter component, is able to grow, albeit at a reduced rate, with GlcNAc as the sole carbon source, suggesting that an alternate GlcNAc uptake pathway(s) exists [38]. We observed that c-di-GMP inhibition of *gbpA* expression did not occur during growth relying on the strictly PTS-dependent carbohydrates sucrose and fructose (Fig. 3), suggesting a dependence of c-di-GMP regulation on carbon source availability and the mechanism of uptake. Limitation of PTS-carbohydrates results in increased cAMP biosynthesis and CRP activation, allowing the bacterium to activate alternate metabolic pathways [30]. Our results suggest that c-di-GMP inhibits *gbpA* transcription when cAMP-CRP levels are elevated. We thus considered the possibility that cAMP-CRP regulates *gbpA* transcription in a mechanism that is influenced by c-di-GMP levels.

To test the role of CRP in regulating *gbpA* in response to c-di-GMP, we generated a *V. cholerae* Δ*crp* mutant and examined it for *gbpA* production. By western blot, the Δ*crp* mutant reproducibly produced ~40 % less GbpA than the parent strain, supporting a role for CRP in upregulating GbpA production (Fig. 4a). The *gbpA* transcript was also reduced by 53 % in the Δ*crp* mutant (Fig. 4b). We next determined the role of CRP in mediating c-di-GMP regulation of *gbpA* by assessing expression in the Δ*crp* mutant bearing pPDE. Unlike in wild-type *V. cholerae* with the control plasmid, when intracellular c-di-GMP levels were lowered by PDE production in the ∆*crp* strain, there was no change in *gbpA* transcript or GbpA protein levels (Fig. 4a and 4b, respectively), indicating that CRP is required for activation of *gbpA* expression in response to decreased c-di-GMP concentrations.

**Fig. 5:**
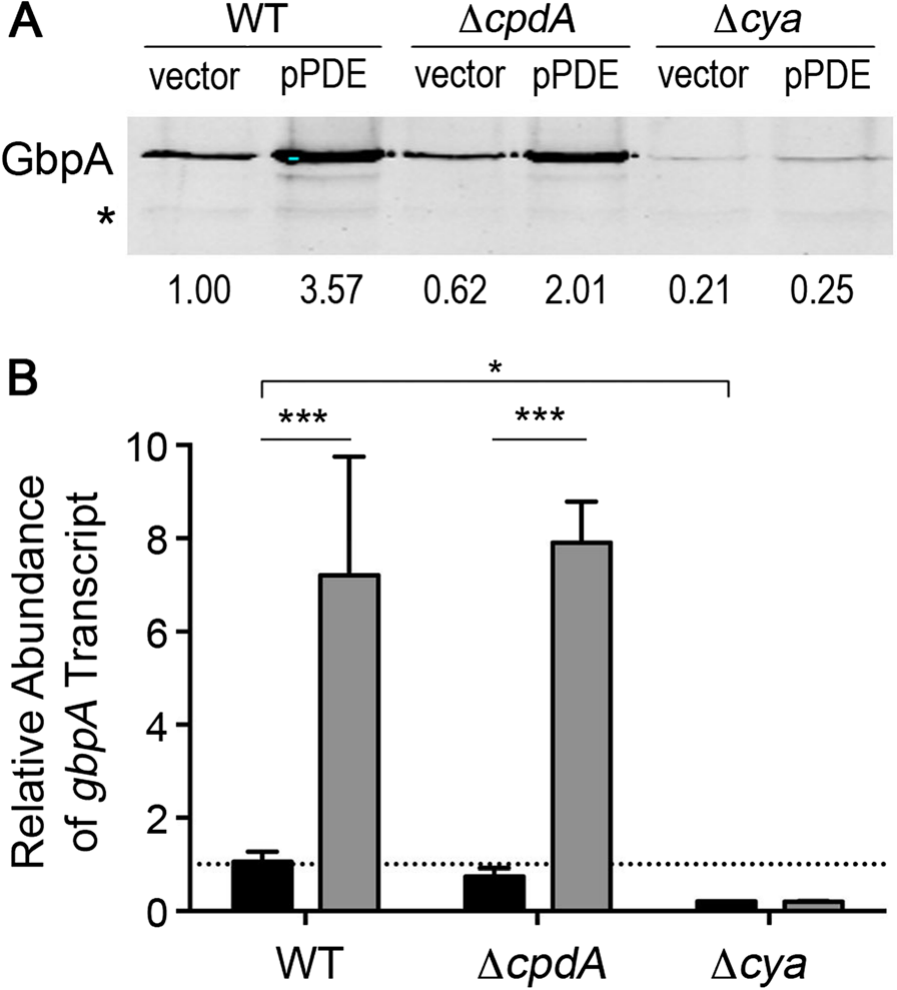
Inactivation of the cAMP-CRP signaling pathway prevents c-di-GMP inhibition of *gbpA* expression. **a** GbpA levels in the supernatants of wild type *V. cholerae*, ∆*cpdA* (constitutively active CRP) and ∆*cya* (constitutively inactive CRP) strains, each with wild type (vector) and reduced levels of c-di-GMP (pPDE), were measured by western blot. PDE gene expression was induced as described in the Methods. The image shown is a representative of three separate experiments. Densitometry analyses were done by comparing the intensities of the GbpA bands to the intensities of a cross-reactive band in the same lane (indicated by an asterisk), then normalizing the value to that of wild type *V. cholerae* with vector. The fold change relative to the wild type is indicated below each lane. **b** qRT-PCR was used to measure the *gbpA* transcript abundance in wild type, ∆*cpdA* and ∆*cya* strains of *V. cholerae,* each with wild type (vector, black bars) and reduced levels of c-di-GMP (pPDE, grey bars). The data were normalized relative to the wild-type containing vector only, using *rpoB* as the reference gene. Shown are the means and standard deviations from at least three independent samples. For the indicated comparisons, *P < 0.05, ***P < 0.001 by unpaired *t*-test

We next evaluated the role of cAMP in the regulation of *gbpA* in *V. cholerae.* We generated a strain with an in-frame deletion of *cya*, which encodes the adenylate cyclase that synthesizes cAMP, yielding a strain that is incapable of CRP activation [59]. The *cya* mutant, like the CRP mutant, produced less GbpA, with an 80 % decrease in protein compared to the wild type (Fig. 5a). A significant 80 % reduction in *gbpA* transcript abundance was also observed in the *cya* mutant using qRT-PCR (Fig. 5b). *V. cholerae* Δ*cya* was then transformed with pPDE or the control vector, allowing us to determine the interplay between cAMP and c-di-GMP in controlling *gbpA* expression. When intracellular c-di-GMP was lowered by PDE production in the ∆*cya* background, there was no change in *gbpA* transcript or GbpA protein levels (Fig. 5a and 5b), mirroring the effect seen with the *crp* mutation.

Additionally, we tested the effect of CRP activation on GbpA production by mutating *cpdA*, which encodes the cAMP phosphodiesterase. In this mutant, cAMP cannot be degraded, and cAMP-CRP complex is constitutively active [54]. In the *cpdA* mutant the levels of *gbpA* transcript and GbpA production were comparable the wild type parent (Fig. 5a and 5b). Reducing c-di-GMP (pPDE) in the *cpdA* mutant increased *gbpA* transcript and GbpA protein levels by 10.5-fold and 3.2-fold, respectively, compared to the same strain with unmodified c-di-GMP (Fig. 5a and 5b). These data further support that cAMP-CRP activates the expression of *gbpA*, and indicate that cAMP and CRP are required for c-di-GMP inhibition of *gbpA* expression.

### CRP production and activity are not directly affected by c-di-GMP

**Fig. 6:**
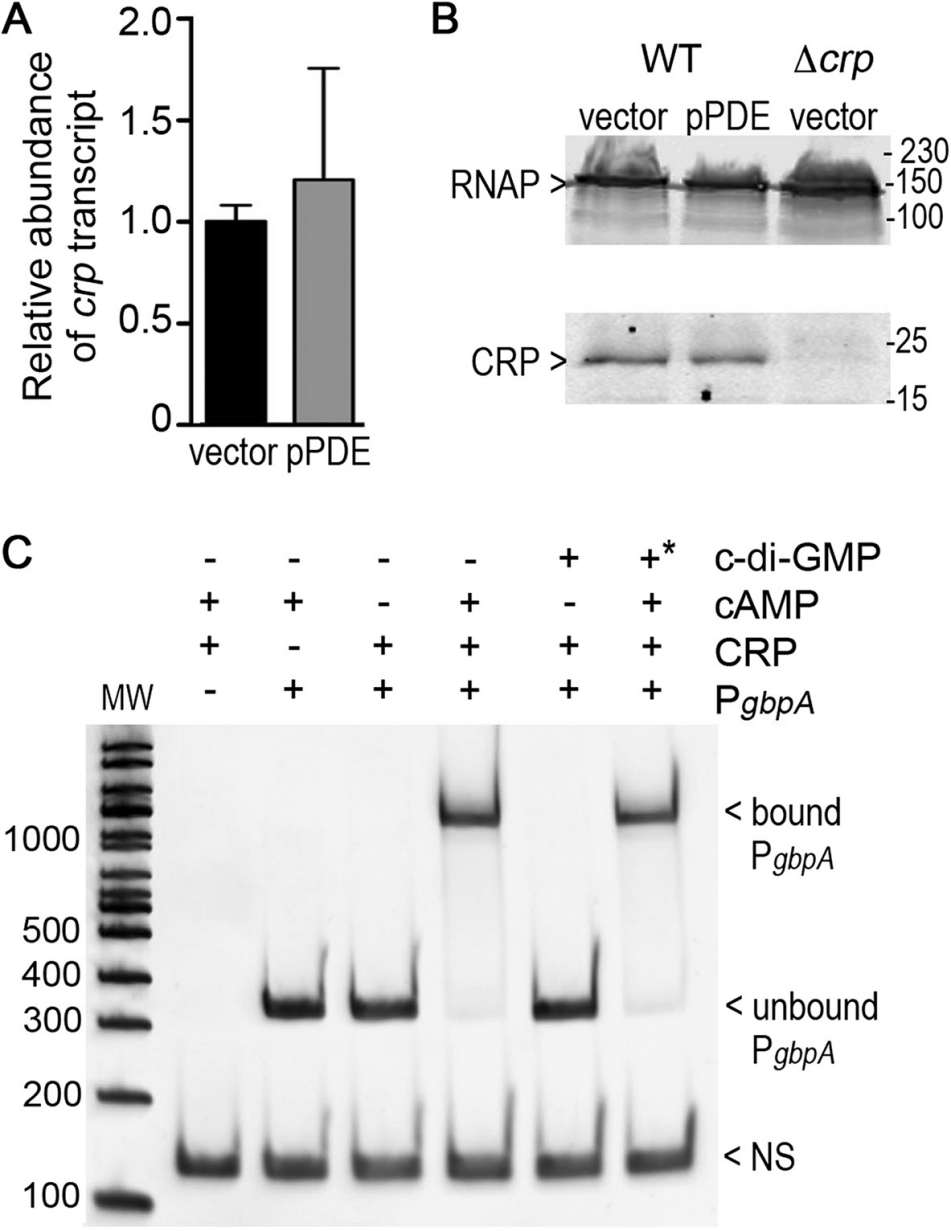
c-di-GMP does not regulate CRP gene transcription, protein stability or DNA binding. **a** Transcript levels for *crp* in *V. cholerae* with wild type (vector) or reduced c-di-GMP (pPDE) were measured by qRT-PCR. PDE gene expression was induced as described in the Methods. The data were normalized relative to the wild-type containing vector only, using *rpoB* as the reference gene. Shown are the means and standard deviations from at three independent samples. **b** CRP protein (23.6 kDa) levels in lysates of *V. cholerae* with wild type (vector) or reduced c-di-GMP (pPDE) were measured by western blot. *V. cholerae* Δ*crp* containing vector was included as a negative control. RNA Polymerase was detected on the same blot as a loading control. The images shown are from a representative of three independent experiments. Densitometry analyses were done by comparing the intensities of the GbpA bands to the intensities of the RNAP band in the same lane, then normalizing the value to that of wild type *V. cholerae* with vector. The fold change relative to the wild type is shown below each lane. **c** Using electrophoretic mobility shift assays, purified recombinant CRP was tested for the ability to bind and shift a DNA fragment encompassing the *gbpA* promoter in the presence or absence of cAMP and/or c-di-GMP. As a control, a non-specific DNA (indicated by “NS”) fragment was added to all binding reactions and was confirmed not to be shifted by CRP. *In the final lane, c-di-GMP was added in 10-fold excess of cAMP

We next sought to understand how c-di-GMP influences CRP-dependent activation of *gbpA* expression; c-di-GMP could regulate *crp* transcription, CRP protein levels or CRP regulatory activity. To assess the effect of c-di-GMP on *crp* expression, *crp* transcript levels were measured in *V. cholerae* with wild type (vector) or reduced c-di-GMP levels (pPDE). Transcript levels of *crp* were not affected by altering c-di-GMP (Fig. 6a), suggesting that c-di-GMP directly or indirectly affects CRP protein levels or activity. The effect of c-di-GMP on CRP protein levels was determined by western blot. Lysates from wild type *V. cholerae* with vector or pPDE, as well as a *crp*-null control strain containing vector, were probed with anti-CRP antibodies. No differences in CRP abundance were apparent in *V. cholerae* with wild type or low c-di-GMP (Fig. 6b).

The identification of a consensus CRP binding site (GTGAGAGCTTGATTCCACATAT) upstream of *gbpA* (and upstream of the Vc1 sequence) using Virtual Footprint software [60] suggests that CRP interacts directly with the *gbpA* promoter. We postulated that c-di-GMP may interfere with the DNA-binding activity of CRP. To test this, we used electrophoretic mobility shift assays (EMSAs) to evaluate the interactions between CRP, c-di-GMP and the *gbpA* promoter. A 293 bp DNA fragment encompassing the *gbpA* promoter and C-terminally tagged CRP were used, and a non-specific 133 bp DNA fragment was included as a negative control in each sample. As observed previously for other promoters [61, 62], CRP alone was unable to bind the *gbpA* promoter fragment, but upon addition of the CRP ligand cAMP, CRP bound and shifted the *gbpA* promoter fragment (Fig. 6c). In contrast, the presence of c-di-GMP did not promote an interaction between CRP and the promoter fragment. Moreover, the addition of 10-fold excess c-d-GMP did not interfere with cAMP-CRP binding to the *gbpA* promoter. Therefore, *in vitro*, c-di-GMP does not influence the binding of cAMP–CRP to the *gbpA* promoter.

### The c-di-GMP and cAMP-CRP signaling pathways act together on the *gbpA* promoter, but not other cAMP-CRP regulatory targets

**Fig. 7:**
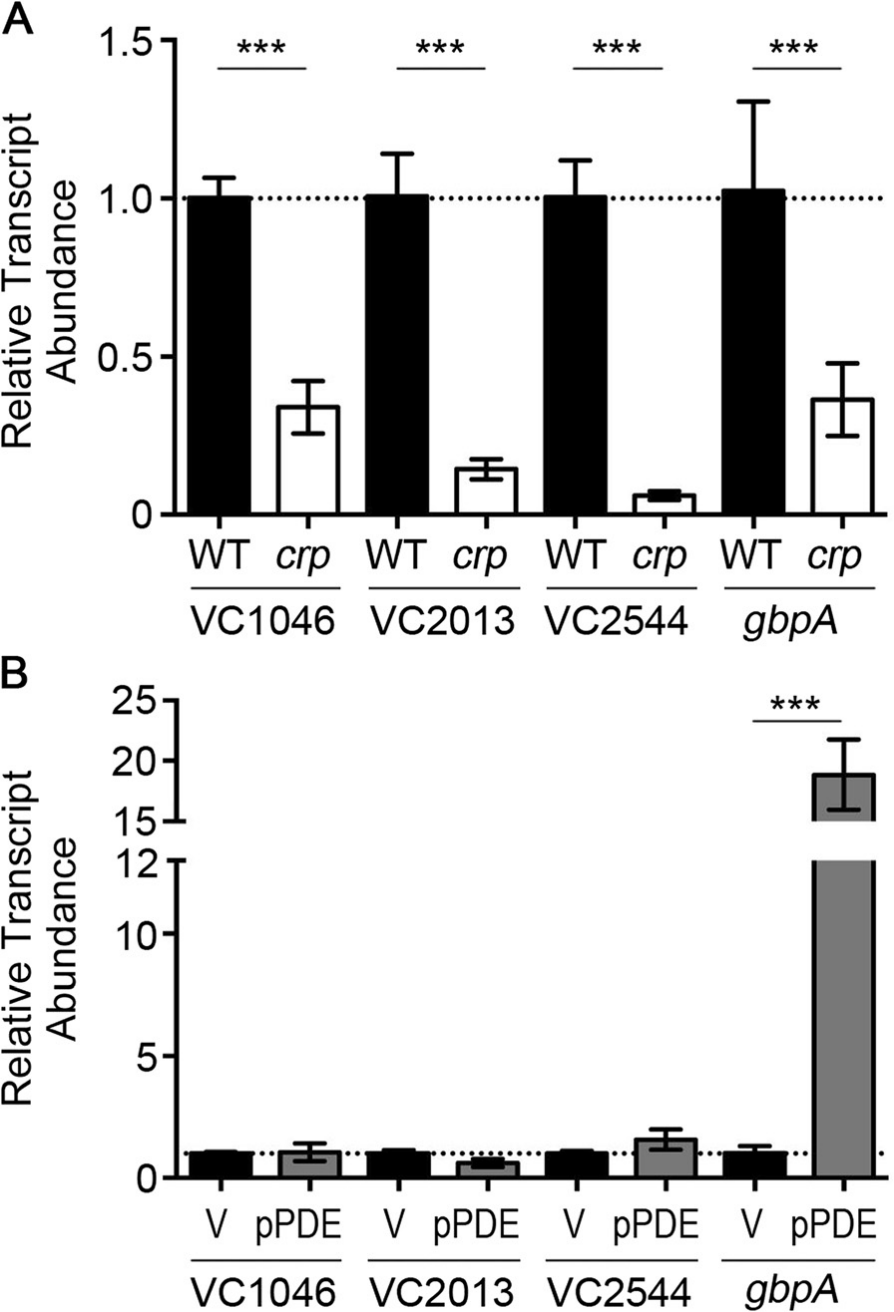
cAMP-CRP specifically impacts *gbpA* expression in response to c-di-GMP. **a** Putative CRP-regulated genes were selected for analysis by qRT-PCR to assess transcript abundance in wild type (grey bars) and Δ*crp* (black bars) strains. The data were normalized relative to the wild-type, using *rpoB* and *gyrA* as the reference genes. **b** The transcript abundance for the genes analyzed in (**a**) was determined for *V. cholerae* with wild type (vector, grey bars) and reduced levels of c-di-GMP (pPDE, white bars). The data were normalized relative to the wild-type containing vector only, using *rpoB* and *gyrA* as the reference genes. **a** and (**b**) Shown are the means and standard deviations from at least three independent samples. For the indicated comparisons, ***P < 0.001 by unpaired *t*-test

To determine if c-di-GMP impacts the regulatory function of CRP, we assessed the regulation of additional CRP regulatory targets by c-di-GMP. We predicted that if c-di-GMP was having a global effect on CRP activity, additional CRP targets would be regulated in a fashion similar to *gbpA*. We tested three metabolic genes that are predicted targets of cAMP-CRP, VC1046, VC2013, and VC2544. All three genes were identified in a transcriptome analysis identifying CRP and cAMP regulated genes [33], and we additionally selected them because they each have a predicted CRP binding site in their promoter (Virtual Footprint) [60]. Our results showed that relative to the wild type parent strain, transcript levels of VC1046, VC2013, and VC2544 were reduced by 65 % or more in the ∆*crp* background, suggesting that these genes are positively regulated by CRP (Fig. 7a). Next, we compared the abundance of these transcripts in wild type *V. cholerae* with vector or pPDE to determine whether manipulation of c-di-GMP affected expression of these genes. Whereas c-di-GMP depletion resulted in an 18.5-fold increase in *gbpA* transcript abundance, we observed no difference in the abundance of these transcripts in the strain with low c-di-GMP, as compared to wild type c-di-GMP levels (Fig. 7b). Together, these results suggest that the effect of low c-di-GMP on CRP activity at the *gbpA* promoter is not a global regulatory effect and that an additional factor mediates the impact of c-di-GMP on *gbpA* transcription initiation.

## Discussion

In response to extracellular stimuli, bacteria manipulate the levels of intracellular second messengers to drive behavioral changes that promote survival. Herein, we describe the combined effects of two nucleotide second messengers, c-di-GMP and cAMP, on transcriptional regulation of *gbpA,* a gene encoding a *V. cholerae* colonization factor produced in aquatic and host environmental conditions. Whereas cAMP promotes *gbpA* transcription via CRP binding to the *gbpA* promoter, c-di-GMP has a negative effect of *gbpA* promoter activity, and cAMP-CRP is epistatic to c-di-GMP regulation . As distinct extracellular cues trigger the c-di-GMP and cAMP signaling pathways, GbpA production may be modulated in response to multiple environmental parameters encountered by *V. cholerae*.

Having determined that c-di-GMP negatively affects *gbpA* promoter activity, we sought to identify the c-di-GMP effector that acts on the *gbpA* promoter. In the process, we determined that CRP activates *gbpA* transcription. The CRP protein binds the *gbpA* promoter in a cAMP-dependent manner *in vitro*, and a *crp* mutant has somewhat decreased production of GbpA. Moreover, in the *crp* mutant, depletion of c-di-GMP did not result in increased *gbpA* expression. The same effect was apparent in a *cya* mutant, which lacks the adenylate cyclase responsible for cAMP synthesis (eliminating the CRP activating signal). The observation that a cAMP phosphodiesterase (*cpdA*) mutant, with constitutively activated CRP, showed the same increase in GbpA upon c-di-GMP depletion as the parental strain indicates that cAMP-CRP promotes *gbpA* transcription, and only under CRP-activating conditions is the inhibitory effect of c-di-GMP on *gbpA* transcription apparent. Consistent with this, c-di-GMP inhibition of *gbpA* expression was observed during growth on carbon sources that do not rely on a PTS for uptake (glucose, maltose and casamino acids)—conditions in which cAMP is produced and CRP is active [31]. In contrast, growth in media with the PTS-dependent sugars sucrose and fructose, whose uptake does not stimulate cAMP production and CRP activation [31], did not reveal an effect of c-di-GMP. Thus, c-di-GMP inhibition of *gbpA* transcription is observable when cAMP-CRP levels are high. That *gbpA* transcription was comparable in the presence of PTS-dependent and independent nutrient sources was a surprising result given that we anticipated that growth with PTS carbohydrates and the resulting low cAMP-CRP would decrease *gbpA* expression. However, it is possible that expression is affected by growth rate, which varies between growth media.

Given the role of CRP in c-di-GMP regulation of *gbpA* expression, we explored the possibility that c-di-GMP influences CRP at various levels. We excluded effects of c-di-GMP on CRP gene transcription and protein abundance. In other bacterial species, c-di-GMP has a direct role in controlling the activity of CRP-like proteins [63, 64]. For example, c-di-GMP has been shown to bind CAP (catabolite activation protein)-like protein CLP from *Xanthomonas campestris*, which inhibits its ability to bind DNA and thus to regulate virulence gene expression [63]. Our data indicate that, while CRP does bind the *gbpA* promoter in a cAMP-dependent manner, c-di-GMP does not affect cAMP-CRP binding*.* Finally, c-di-GMP did not affect CRP regulatory function, because c-di-GMP did not broadly affect the expression of other CRP-regulated genes. Together these findings indicate that c-di-GMP does not regulate CRP, but regulates another effector that co-regulates *gbpA* expression.

Several other potential mediators of c-di-GMP inhibition of *gbpA* transcription were considered. First, we examined the role of NagC. Not only was NagC the only known regulator of *gbpA* expression [38], the NagC and CRP regulons were previously linked in *E. coli*, in which the NagC and CRP orthologues co-regulate the expression of genes in the chitobiose operon [65]. While our results support previous reports indicating that NagC negatively regulates *gbpA*, this GlcNAc-responsive regulator had no impact on c-di-GMP regulation of *gbpA* expression. GlcNAc was previously suggested to be a PTS-dependent sugar in *V. cholerae*, in which case growth with GlcNAc as the sole carbon source would not be expected to activate the cAMP-CRP pathway [31]. However, upregulation of *gbpA* was observed upon c-di-GMP depletion during growth with GlcNAc, mirroring the results obtained during growth with PTS-independent nutrients. One possible explanation for these results is that *gbpA* is regulated via multiple mechanisms during growth in GlcNAc (cAMP-CRP, NagC, c-di-GMP, possibly others), making the net *gbpA* transcription level difficult to predict. Alternatively, GlcNAc, like glucose, may not strictly rely on a PTS for uptake in *V. cholerae*. Indeed, although certain PTS components are essential for GlcNAc utilization in *V. cholerae*, a strain deficient in the EIIB^GlcNAc^ transporter (NagE) retains the ability to grow with GlcNAc as the sole carbon source [38], suggesting that an additional GlcNAc transporter exists in this bacterium. Thus, an alternative pathway for GlcNAc transport would alleviate any impact on the cAMP-CRP pathway.

We directly evaluated three previously defined transcription factors known to bind c-di-GMP in *V. cholerae*, VpsT, VpsR and FlrA, as potential mediators of *gbpA* inhibition by c-di-GMP. These regulators were compelling candidates, because *gbpA* (VCA0811) appeared in transcriptional profiling studies of the respective mutants, with VpsT and VpsR suggested to act as activators and FlrA acting as a repressor [20, 58]. However, none of these transcription factors significantly altered *gbpA* expression or c-di-GMP regulation of *gbpA*. The effector that senses c-di-GMP and impinges on *gbpA* expression remains unidentified.

It is possible that carbon source influences c-di-GMP levels in *V. cholerae*. Earlier studies showed cross-talk between cAMP and c-di-GMP signaling pathways in *V. cholerae.* Fong *et al.* have demonstrated that cAMP-CRP signaling can impact c-di-GMP production by repressing the expression of the DGC *cdgA*, and accordingly, a *crp* mutant behaves like a strain with elevated c-di-GMP levels [33]. To our knowledge, our findings are the first to link the regulatory effects of both cAMP and c-di-GMP at a single promoter. Increasing cAMP levels by inactivating the phosphodiesterase CpdA did not alter *gbpA* expression, suggesting that when CRP is maximally active, c-di-GMP can still inhibit CRP activation of *gbpA*. We speculate that direct interplay between cAMP-CRP and a c-di-GMP regulated factor may impact *gbpA* transcription such that maximal CRP-dependent activation of *gbpA* is apparent under conditions in which cAMP is abundant and c-di-GMP is low. Alternatively, the two regulatory events may be independent, and the effect of c-di-GMP is only observable when cAMP-CRP is present. Additional studies are needed to determine how nutrient availability, cAMP-CRP and c-di-GMP interact to control *gbpA* expression, and perhaps expression of other genes.

Previous studies have demonstrated a link between the available carbon source and surface colonization by *V. cholerae*. The ability to respond to PTS carbohydrates is important for binding to chitin, chitin degradation, and chitin-induced competence [54, 66]. The presence of PTS sugars, mutation of the adenylate cyclase gene *cyaA*, and mutation of *crp*, each of which reduce or eliminate cAMP-CRP, diminish the ability of *V. cholerae* to interact with chitin [66]. As GbpA also plays a role in colonization of chitin and intestinal surfaces by *V. cholerae* [35, 36], it is tempting to speculate that cAMP-CRP links *gbpA* expression with expression of genes encoding chitin utilization and chitin-induced competence components. In addition, in a germ-free mouse model, *V. cholerae* requires a functional PTS system to persist in the intestine, indicating that this bacterium relies on PTS carbohydrates during infection [31]. cAMP-CRP also influences expression of numerous other virulence factors of *V. cholerae* [32, 59, 67, 68]. Thus GbpA is part of the larger cAMP-CRP regulated program central to surface colonization by *V. cholerae*.

Genetic evidence suggests that *V. cholerae* modulates intracellular c-di-GMP levels during transitions between its native aquatic environment and the host intestine. Biofilm formation is positively regulated by c-di-GMP, which may enhance *V. cholerae* survival on chitin and other aquatic surfaces [18, 22, 50]. Indeed, c-di-GMP regulates the production of at least one other chitin binding protein, the hemagglutinin FrhA, and has been demonstrated to promote attachment of *V. cholerae* to chitin beads [20]. Reduction of c-di-GMP is required to promote bacterial motility and increase expression of virulence factors [21, 49, 69–71], and there is evidence suggesting that *V. cholerae* may increase c-di-GMP at later stages of infection [72]. Dysregulation of c-di-GMP signaling likely affects the ability of *V. cholerae* to persist in and transition between these environments.

The data presented here and in previous reports point to an exceedingly complex regulation of GbpA. In addition to derepression of *gbpA* by NagC in response to GlcNAc, we herein show that high c-di-GMP interferes with cAMP-CRP activation of *gbpA* transcription. Thus, the net production of GbpA would depend upon the levels of intracellular cAMP and the c-di-GMP. Another layer of GbpA regulation occurs through post-translational hydrolysis of GbpA by two quorum sensing regulated proteases, HapA and PrtV [73]. Cell density-dependent hydrolysis of GbpA may also be linked to second messenger levels. The quorum sensing pathway of *V. cholerae* influences c-di-GMP levels at least in part through regulation of c-di-GMP metabolism genes [74, 75]. Quorum sensing is also linked to the cAMP-CRP signaling pathway in *V. cholerae*. CRP impacts the production of the quorum sensing regulator HapR, as well as synthesis of cholera autoinducer 1 [32, 62, 76]. Thus, the c-di-GMP, cAMP and quorum sensing signaling networks are intricately intertwined, leading to complex regulation of GbpA production.

## Conclusions

In sum, the transcriptional, post-transcriptional and post-translational regulation of GbpA may allow *V. cholerae* to fine-tune GbpA production in response to changes in intracellular c-di-GMP concentrations. Importantly, numerous extracellular signals that impact intracellular c-di-GMP and cAMP levels could regulate GbpA production, influencing the ability of *V. cholerae* to colonize aquatic and host surfaces.

## Additional files

Additional file 1:
**Supplemental Tables S1-S3: (S1) Strains used in this study, (S2) Plasmids used in this study, and (S3) Primers used in this study.** (PDF 306 kb) Additional file 2:
**Supplemental Figures S1 and S2: (S1) Manipulation of c-di-GMP in** ***V. cholerae*** **following ectopic expression of phosphodiesterase genes, and (S2) Growth of** ***V. cholerae*** **reporter strains on various carbon sources.** (PDF 252 kb)

## Abbreviations

c-di-GMP: 3′-5′ cyclic diguanylic acid, cyclic diguanylate
cAMP: Cyclic adenosine monophosphate
cGMP: Cyclic guanosine monophosphate
(p)ppGpp: Guanosine pentaphosphate or tetraphosphate
LB: Luria-Bertani
MM: M9 minimal medium
GlcNAc: N-acetylglucosamine
Sm: Streptomycin
Amp: Ampicillin
Cm: Chloramphenicol
DGC: Diguanylate cyclase
PDE: Phosphodiesterase
AC: Adenylate cyclase
CRP: cAMP receptor protein
PTS: Phosphoenolpyruvate-carbohydrate phosphotransferase transport system
EPS: Exopolysaccharide
UTR: Untranslated region
qRT-PCR: Quantitative real-time polymerase chain reaction
