# Supplementary material for: Two nucleotide second messengers regulate the production of the Vibrio cholerae colonization factor GbpA
Source: BMC Microbiol. 2015 Aug 19;15:166. doi: 10.1186/s12866-015-0506-5 (PMC4545359; doi:10.1186/s12866-015-0506-5)
Supplement: Additional file 1: — Supplemental Tables S1-S3: (S1) Strains used in this study, (S2) Plasmids used in this study, and (S3) Primers used in this study. (PDF 306 kb) [file 12866_2015_506_MOESM1_ESM.pdf]

SUPPLEMENTAL TABLES

Two nucleotide second messengers regulate the production of the *Vibrio cholerae* colonization factor GbpA

Running title: c-di-GMP and cAMP regulation of GbpA

Ankunda T. Kariisa, Alyssa Grube, and Rita Tamayo*

Department of Microbiology and Immunology, University of North Carolina Chapel Hill, North Carolina, USA

*Corresponding Author Contact Information:

Department of Microbiology and Immunology CB# 7290

University of North Carolina Chapel Hill, North Carolina, USA,

Phone: (919) 843-2864

Fax: (919) 962-8103

E-mail: rita_tamayo@med.unc.edu

Table S1. Strains used in this study.

Strain	Strain Description	Reference
Escherichia coli		
DH5α	F⁻ φ80*lacZ* ΔM15 Δ(*lacZYA-argF*)*U169 recA1 endA1 hsdR17* (rK-, mK+) *phoA supE44 thi-1 gvrA96 relA1 λ- tonA*	Invitrogen; [1]
DH5αλpir	F⁻ Δ(*lacZYA-argF*)*U169 recA1 endA1 hsdR17 supE44 thi-1 gvrA96 relA1 λpir*	[1]
SM10λpir	*thi thr leu tonA lacY supE recA*::RPA-2-Te::Mu *λpir*R6K, Km^r	[2]
Vibrio cholerae		
C6706	Wild type, O1 El Tor	[3]
C6706 Δ*gbpA*	C6706 with in-frame deletion in *gbpA* (VCA0811)	This study
C6706 Δ*lacZ*	C6706 with in-frame deletion in *lacZ*	[4]
C6706 Δ*lacZ* pP_{gbpA}-Vc1-*lacZ*	C6706 Δ*lacZ* bearing translational reporter of the *gbpA* promoter and 5' untranslated region (UTR) to *E. coli lacZ*	This study
C6706 Δ*lacZ* pP_{gbpA}-ΔVc1-*lacZ*	C6706 Δ*lacZ* bearing translational reporter of the *gbpA* promoter and 5' UTR, with Vc1 deleted, to *E. coli lacZ*	This study
C6706 Δ*lacZ* pP_{gbpA}-Vc1-*lacZ* pBAD33	C6706 Δ*lacZ* bearing translational reporter of the *gbpA* promoter and 5' untranslated region (UTR) to *E. coli lacZ*, with pBAD33	This study
C6706 Δ*lacZ* pP_{gbpA}-Vc1-*lacZ* pPDE	C6706 Δ*lacZ* bearing translational reporter of the *gbpA* promoter and 5' untranslated region (UTR) to *E. coli lacZ*, with pPDE	This study
C6706 pBAD33	C6706 bearing vector pBAD33	This study
C6706 pPDE	C6706 pBAD33::*vieA* ("pPDE") (encodes EAL domain PDE)	This study
C6706 $pPDE^{mut}$	C6706 pBAD33::*vieAE170A* ("$pPDE^{mut}$") (encodes enzymatically inactive PDE)	This study
C6706 pVC1592	C6706 pBAD33::VC1592 (encodes EAL domain PDE)	This study
C6706 ΔVc1	C6706 with in-frame deletion of Vc1 element upstream of *gbpA* open reading frame	This study
C6706 ΔVc1 pBAD33	C6706 ΔVc1 bearing vector pBAD33	This study
C6706 ΔVc1 pPDE	C6706 ΔVc1 with pBAD33::*vieA* (pPDE)	This study
C6706 Δ*nagC*	C6706 with in-frame deletion of *nagC*	This study
C6706 Δ*nagC* pBAD33	C6706 Δ*nagC* with vector pBAD33	This study
C6706 Δ*nagC* pPDE	C6706 Δ*nagC* with pBAD33::*vieA* (pPDE)	This study
C6706 Δ*crp*	C6706 with in-frame deletion of *crp*	This study
C6706 Δ*crp* pBAD33	C6706 Δ*crp* with vector pBAD33	This study
C6706 Δ*crp* pPDE	C6706 Δ*crp* with pBAD33::*vieA* (pPDE)	This study
C6706 Δ*cpdA*	C6706 with in-frame deletion of *cpdA*	This study
C6706 Δ*cpdA* pBAD33	C6706 Δ*cpdA* with vector pBAD33	This study
C6706 Δ*cpdA* pPDE	C6706 Δ*cpdA* with pBAD33::*vieA* (pPDE)	This study
C6706 Δ*cya*	C6706 with in-frame deletion of *cya*	This study
C6706 Δ*cya* pBAD33	C6706 Δ*cya* with vector pBAD33	This study
C6706 Δ*cya* pPDE	C6706 Δ*cya* with pBAD33::*vieA* (pPDE)	This study

C6706 Δ*vpsT*	C6706 with in-frame deletion of *vpsT*	This study
C6706 Δ*vpsT* pBAD33	C6706 Δ*vpsT* with vector pBAD33	This study
C6706 Δ*vpsT* pPDE	C6706 Δ*vpsT* with pBAD33::*vieA* (pPDE)	This study
C6706 Δ*vpsR*	C6706 with in-frame deletion of Δ*vpsR*	[5]
C6706 Δ*vpsR* pBAD33	C6706 Δ*vpsR* with vector pBAD33	This study
C6706 Δ*vpsR* pPDE	C6706 Δ*vpsR* with pBAD33::*vieA* (pPDE)	This study
C6706 Δ*flrA*	C6706 with in-frame deletion of Δ*flrA*	This study
C6706 Δ*flrA* pBAD33	C6706 Δ*flrA* with vector pBAD33	This study
C6706 Δ*flrA* pPDE	C6706 Δ*flrA* with pBAD33::*vieA* (pPDE)	This study

Table S2. Plasmids used in this study.

Plasmid Name	Plasmid Description	Reference
pP*lac*thiM#2-*lacZ*	The TPP riboswitch encoded in the 5' UTR of *E. coli thiM* was mutated to function as an ON switch and cloned into pP*lac*-*lacZ*. Origin of replication *colEI.* Amp^R	[6]
pP_{gbpA}-Vc1-*lacZ*	*gbpA* promoter and 5'UTR cloned into plasmid pP*lac*thiM#2-*lacZ*; translational fusion	This study
pP_{gbpA}-ΔVc1-*lacZ*	*gbpA* promoter and 5'UTR, minus the Vc1 sequence (Δ 17-202 bp) cloned into plasmid pP*lac*thiM#2-*lacZ*; translational fusion	This study
pBAD33	Expression vector, P_{ara} promoter, Cm^R	[7]
pPDE	pBAD33::*vieA-His6*	[8]
$pPDE^{mut}$	pBAD33::*vieA-E170A,* allele encodes inactive PDE	[8]
pVC1592	pBAD33::VC1592 (EAL domain PDE gene)	This study
pBAD33::*crp*-his6	Expression vector for purification of CRP	This study
pCVD442	*oriR6K* plasmid with a polylinker, *mobRP4*, *bla*, and *sacB*	[9]
pCVD442::Δ*gbpA*	Allelic exchange vector for in-frame deletion of *gbpA*	This study
pCVD442::ΔVc1	Allelic exchange vector for in-frame deletion of Vc1	This study
pCVD442::Δ*nagC*	Allelic exchange vector for in-frame deletion of *nagC*	This study
pCVD442::Δ*crp*	Allelic exchange vector for in-frame deletion of *crp* (VC2614)	This study
pCVD442::Δ*cya*	Allelic exchange vector for in-frame deletion of *cya* (VC0122)	This study
pCVD442::Δ*cpdA*	Allelic exchange vector for in-frame deletion of *cpdA*	This study
pCVD442::Δ*vpsT*	Allelic exchange vector for in-frame deletion of *vpsT*	This study
pCVD442::Δ*vpsR*	Allelic exchange vector for in-frame deletion of *vpsR*	[5]
pCVD442::Δ*flrA*	Allelic exchange vector for in-frame deletion of *flrA*	This study

Table S3. Primers used in this study.

Primer Name	Oligonucleotide sequence (5' to 3') *	Reference
pCVDseqF	CTGTTGCATGGGCATAAAGTTGCC	This study
pCVDseqR	ACACAGGAACACTTAACGGCTGAC	This study
Vc1F1	CCTCTAGATACTCGTCAGGTCTTTGG	This study
Vc1R1	TTGCATGCTTGCCAAAGTGTGACCGAGAG	This study
Vc1F2	TTGCATGCCAGCTAACAAAGAAGAGTCTGTG	This study
Vc1R2	AAGAGCTCCGAGTGTACACGGTATCG	This study
gbpAF1	TTGCATGCTACTCGTCAGGTCTTTGG	This study
gbpAR1	TTGGTACCCATCACAGACTCTTCTTTG	This study
gbpAF2	TTGGTACCTAAGTTATCCTCCCTCTTAC	This study
gbpAR2	TTGAGCTCTTTCTCTGGATGGGAGTC	This study
gbpAF0	GCAAACGGTAGCAAGAAG	This study
nagCF1	GAGCATGCCTGACGATACGATGATTGATAC	This study
nagCR1	GACCATGGCATTATCAATTCTGCTCGTATTGTC	This study
nagCF2	GACCATGGGAAGACTGAGCTTTTTAGTTAAGC	This study
nagCR2	GAGAGCTCGAGCATCATCCCTAAGATCAG	This study
nagCF0	CGATACCATGCACAAAGC	This study
crpF1	CCTCTAGATTTATGAAGGCTTACACGGC	This study
crpR1	CCGCATGCCATAATAATCTCACTTCCTCTGCAG	This study
crpF2	CCGCATGCTAAGTGCCCCGATAACCC	This study
crpR2	CCGAGCTCTTGAACATCCCGATCCTTTG	This study
crpF0	CCCTACTTACTGGCGATGAT	This study
cyaF1	CCGAGCTCCATTGACGGCGTAAACTG	This study
cyaR1	CCGAATTCCAAGTTTGCTTCCCTGATATG	This study
cyaF2	CCGAATTCTAACTCGTTGACGTCTCAG	This study
cyaR2	CCTCTAGACATAAAGCGGTGTACCGTAC	This study
cyaF0	CAAGCTTACTTAGGCGAGTC	This study
cpdAF1	CCTCTAGAGCTGACTGTAGGCAGAATTG	This study
cpdAR1	GGGCATGCCAAAATCGGTAAACCTAACTCTG	This study
cpdAF2	GGGCATGCTGATATGACTGCTCGTCCTG	This study
cpdAR2	AAGAGCTCGTCTAAGGGCAACATAGTCTG	This study
cpdAF0	GTCGAGCGGGAAATGTTTG	This study
vpsTF1	CCGCATGCGCGAAGTTTCACGTACTCG	This study
vpsTR1	CCGGTACCCATTTCACCCCTCCTAACAC	This study
vpsTF2	CCGGTACCTAATTCGTTGTGTAATGTCTCTTCG	This study
vpsTR2	CCGAGCTCCATCCCACACATACCAACC	This study
vpsTF0	CGATGATATCTTGGCTCAACTC	This study
vpsTF1	TGCATGCCTACAACCCAAATCACGC	This study
vpsTR1	AATCAGCAAAACTTACATGAACCTATATTCCTT	This study
vpsTF2	GAATATAGGTTCATGTAAGTTTTGCTGATTTAC	This study
vpsTR2	TTTCTAGAGGTAAACTCAAGCCGATT	This study
vpsTF0	CTCTGTGGCGTTAGAAG	This study
FLRAF1	CCGCATGCCAACTTGTGGAACAGATGCAG	This study

FLRAR1	CCGGTACCCTGCATAGGTGAGATTATTTGCC	This study
FLRAF2	CCGGTACCAACATGCAACGCTAGGG	This study
FLRAR2	CCGAGCTCTCGACCATAGGCATAAATTCG	This study
flrAF0	GTTAGGCTATTTGGCCGAG	This study
RPB2F	CTGTCTCAAGCCGGTTACAA	[10]
RPB2R	TTTCTACCAGTGCAGAGATGC	[10]
gyrAqF	AATGTGCTGGGCAACGACTG	This study
gyrAqR	GAGCCAAAGTTACCTTGGCC	This study
gbpAqF	CAGTGGATTAGCGTATGGACAC	This study
gbpAqR	GTATTGAATCGCGCCACAGT	This study
crpqF	GGTGAGAAAGCGGAAACGCTGTACTA	This study
crpqR	CTTCAAACAAGCCAAGCTCACCGA	This study
VC1046qF	GCCTTGATCGAGCGGATTAT	This study
VC1046qR	TCACAGGCGATGATGTTGAG	This study
VC2013qF	CTATCGTCGGTTACGGCATTAT	This study
VC2013qR	ACCCAGATATTCAGGCAGTTG	This study
VC2544qF	TTCGCTTGGCACCTTCTATC	This study
VC2544qR	GTCCCTCTTCTGGCACATTT	This study
CRPeF	CCGAATTCGCAGAGGAAGTGAGATTATTATGG	This study
CRPeH6R	CCGTCGACTTA*ATGGTGATGGTGATGGTG*GCGAGTGCCGTAAACCA	This study
VC1592eF	CCGAGCTCTTTAGGATACATTTTTATGAATCACATCCACCCC	This study
VC1592eH6R	AACTGCAGCTA*ATGATGATGATGATGATG*TAACGCAGACATACGTGG	This study

***** The underlined sequences represent restriction enzyme recognition sites. Italicized sequences encode a 6-histidine tag.

References

1. Hanahan D: **Studies on transformation of *Escherichia coli* with plasmids.** *J Mol Biol* 1983, **166**(4):557-80.

2. Miller VL, Mekalanos JJ: **A novel suicide vector and its use in construction of insertion mutations: osmoregulation of outer membrane proteins and virulence determinants in *Vibrio cholerae* requires *toxR*.** *J Bacteriol* 1988, **170**(6):2575-83.

3. Thelin KH, Taylor RK: **Toxin-coregulated pilus, but not mannose-sensitive hemagglutinin, is required for colonization by *Vibrio cholerae* O1 El Tor biotype and O139 strains.** *Infect Immun* 1996, **64**(7):2853-6.

4. Tamayo R, Patimalla B, Camilli A: **Growth in a biofilm induces a hyperinfectious phenotype in *Vibrio cholerae*.** *Infect Immun* 2010, **78**(8):3560-3569.

5. Tamayo R, Schild S, Pratt JT, Camilli A: **Role of cyclic di-GMP during el tor biotype *Vibrio cholerae* infection: characterization of the in vivo-induced cyclic di-GMP phosphodiesterase CdpA.** *Infect Immun* 2008, **76**(4):1617-27.

6. Nomura Y, Yokobayashi Y: **Reengineering a natural riboswitch by dual genetic selection.** *J Am Chem Soc* 2007, **129**(45):13814-13815.

7. Guzman LM, Belin D, Carson MJ, Beckwith J: **Tight regulation, modulation, and high-level expression by vectors containing the arabinose PBAD promoter.** *J Bacteriol* 1995, **177**(14):4121-4130.

8. Tischler AD, Camilli A: **Cyclic diguanylate (c-di-GMP) regulates *Vibrio cholerae* biofilm formation.** *Mol Microbiol* 2004, **53**(3):857-69.

9. Donnenberg MS, Kaper JB: **Construction of an *eae* deletion mutant of enteropathogenic *Escherichia coli* by using a positive-selection suicide vector.** *Infect Immun* 1991, **59**(12):4310-7.

10. Quinones M, Kimsey HH, Waldor MK: **LexA cleavage is required for CTX prophage induction.** *Mol Cell* 2005, **17**(2):291-300.
