# Supplementary material for: Two nucleotide second messengers regulate the production of the Vibrio cholerae colonization factor GbpA
Source: BMC Microbiol. 2015 Aug 19;15:166. doi: 10.1186/s12866-015-0506-5 (PMC4545359; doi:10.1186/s12866-015-0506-5)
Supplement: Additional file 2: — Supplemental Figures S1 and S2: (S1) Manipulation of c-di-GMP in V. cholerae following ectopic expression of phosphodiesterase genes, and (S2) Growth of V. cholerae reporter strains on various carbon sources. (PDF 252 kb) [file 12866_2015_506_MOESM2_ESM.docx]

**SUPPLEMENTAL FIGURES**

**Two nucleotide second messengers regulate the production of the *Vibrio cholerae* colonization factor GbpA**

*Running title: c-di-GMP and cAMP regulation of GbpA*

Ankunda T. Kariisa, Alyssa Grube, and Rita Tamayo^*^

Department of Microbiology and Immunology, University of North Carolina Chapel Hill, North Carolina, USA

*Corresponding Author Contact Information:

Department of Microbiology and Immunology CB# 7290

University of North Carolina Chapel Hill, North Carolina, USA,

Phone: (919) 843-2864

Fax: (919) 962-8103

E-mail: rita_tamayo@med.unc.edu


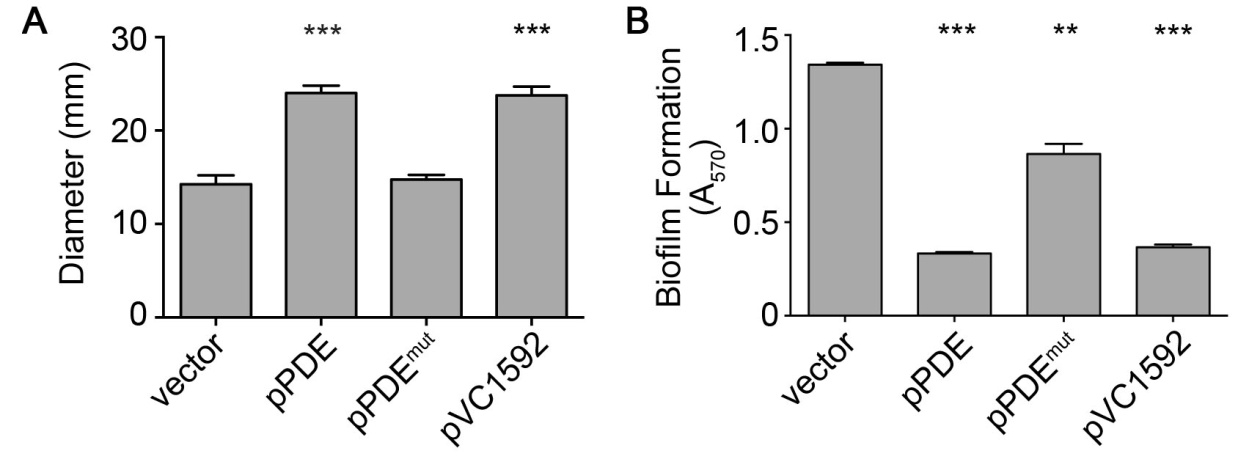


**Figure S1.** Manipulation of c-di-GMP in *V. cholerae* following ectopic expression of phosphodiesterase genes. (A) Motility of *V. cholerae* expressing each PDE gene was assayed in soft agar medium as described previously [1]. *V. cholerae* with vector only served as a control. Strains were grown overnight (16-18 hours) on LB + 1.5% agar at 37 °C. Single colonies were inoculated into motility plates (1% tryptone, 0.5% NaCl, 0.3% agar) supplemented with 0.2% (w/v) L-arabinose to induce gene expression. Motility plates were incubated for 20 hours at room temperature, and diameters of growth were measured. Shown are means and standard deviations for four independent experiments. (B) Biofilm formation by *V. cholerae* producing each of the indicated proteins was assessed as described previously [2, 3]. Each strain was grown in LB broth with 0.2% L-arabinose (to induce gene expression) in 13 mm diameter borosilicate glass tubes at room temperature (~23 ^o^C) for 24 hours. Unattached cells were removed, and the remaining biofilms were washed with water and stained with 0.1% (w/v) crystal violet. After the stained biofilms were washed with water, the stain was solubilized in 2 ml of 50% v/v ethanol. Biofilm formation was determined by measuring the absorbance at 540 nm. Three independent experiments were performed, and the means and standard deviations are shown. (A and B) pPDE = pBAD33::*vieA*; pPDE^mut^ = pBAD33::*vieA*-E170A (encodes inactive VieA); pVC1592 = pBAD33::VC1592 (encodes alternative c-di-GMP PDE). ** p < 0.01, p < 0.001, by student’s t-test compared to *V. cholerae* with vector.


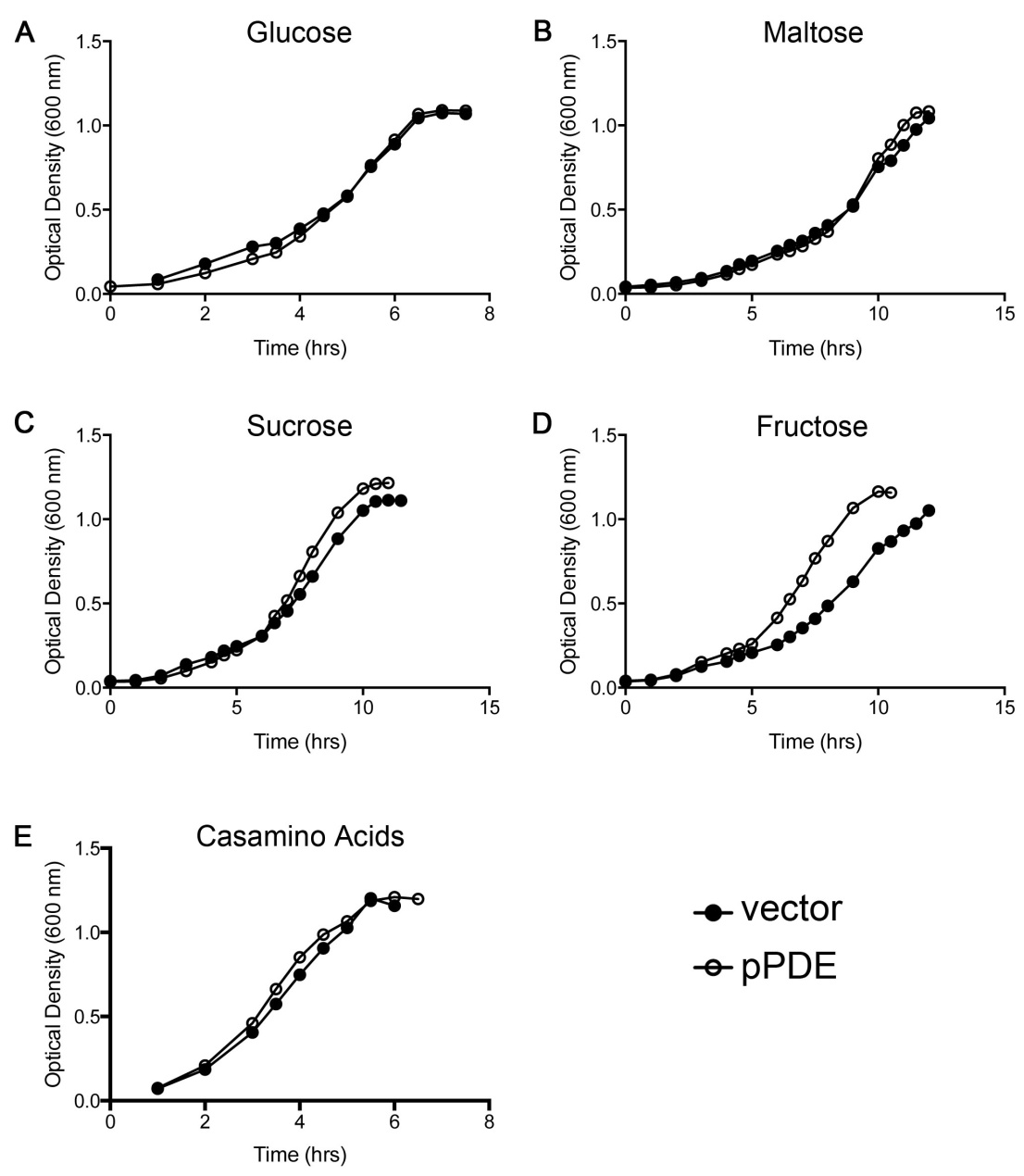


**Figure S2.** Growth of *V. cholerae* reporter strains on various carbon sources. *V. cholerae* containing the pP*_gbpA_*-Vc1-*lacZ* reporter plasmid, with either vector (closed circles) or pPDE (open circles), were grown at 37 ^o^C with aeration in M9 minimal media supplemented with 0.2% L-arabinose (to induce gene expression) and with 0.5% (w/v) of the following: (A) glucose, (B) maltose, (C) sucrose, (D) fructose or (E) casamino acids. To maintain the plasmids, 10μg/ml chloramphenicol and 50 μg/ml ampicillin were included. The optical densities (600 nm) of the cultures were measured hourly. For β-galactosidase assays, qRT-PCR and western blots in this study, samples were collected at mid-exponential phase (OD_600_ 0.45-0.6).

**References**

1. Pratt JT, Tamayo R, Tischler AD, Camilli A: **PilZ domain proteins bind cyclic diguanylate and regulate diverse processes in *Vibrio cholerae*.** *J Biol Chem* 2007, **282**(17):12860-70.

2. Lauriano CM, Ghosh C, Correa NE, Klose KE: **The sodium-driven flagellar motor controls exopolysaccharide expression in *Vibrio cholerae*.** *J Bacteriol* 2004, **186**(15):4864-74.

3. Tamayo R, Patimalla B, Camilli A: **Growth in a biofilm induces a hyperinfectious phenotype in *Vibrio cholerae*.** *Infect Immun* 2010, **78**(8):3560-3569.
